# Posterior decompression and spinous process reconstruction for lumbar spinal stenosis in a pediatric patient with achondroplasia: a case report

**DOI:** 10.1186/s13256-025-05611-6

**Published:** 2025-12-07

**Authors:** Kenta Kudo, Katsunori Fukutake, Takashi Dezawa, Akinori Tani, Kazumasa Nakamura, Keiji Hasegawa, Hiroshi Takahashi, Akihito Wada

**Affiliations:** Department of Orthopedic Surgery, Toho University Omori Medical Center, 6-11-1, Omori-Nishi, Ota-Ku, Tokyo, 143-8541 Japan

**Keywords:** Achondroplasia, Lumbar spinal canal stenosis, Laminotomy with spinous process reconstruction

## Abstract

**Background:**

Achondroplasia is the most prevalent form of skeletal dysplasia and is characterized by rhizomelia, short stature, and distinctive facial features. Achondroplasia is frequently accompanied by spinal canal stenosis because of the distinctive morphology of the spine. For pediatric lumbar spinal canal stenosis, a meticulously constructed surgical plan is needed to prevent complications such as the development of thoracolumbar kyphosis.

**Case presentation:**

An 11-year-old Asian boy with achondroplasia presented with bilateral lower limb numbness and intermittent claudication, which limited his walking distance to 100 m. Imaging revealed multilevel lumbar spinal canal stenosis from T12 to S1, with the most stenosis at the L4/5 level. Laminotomy with spinous process reconstruction using mini plates was performed to preserve the midline posterior tension band. The patient’s postoperative course was uneventful, with immediate symptom resolution and no symptoms of recurrence or signs of kyphotic deformity at the 2-year follow-up.

**Discussion:**

Achondroplasia-associated lumbar spinal canal stenosis arises from anatomical constraints, such as shortened pedicles and short interpedicular distances. Surgical intervention must provide effective decompression without increasing the risk of postoperative thoracolumbar kyphosis. This case highlights the importance of preserving posterior elements that are responsible for maintaining spinal stability and the pertinence of avoiding extensive fixation, particularly in pediatric patients with achondroplasia.

**Conclusion:**

This case demonstrates that, in patients with achondroplasia, laminotomy with spinous process reconstruction can effectively address lumbar spinal canal stenosis and preserve posterior elements with a minimal risk of complications. Long-term follow-up remains crucial for monitoring the potential development of thoracolumbar kyphosis.

## Background

Achondroplasia (ACH) represents the most prevalent form of skeletal dysplasia and is characterized by rhizomelia, short stature resulting from impaired endochondral ossification, and distinctive facial features (for example, frontal bossing and a depressed nasal bridge) [1–3]. The prevalence of ACH is estimated to range from 1 in 10,000 to 30,000 individuals, with more than 98% of patients presenting a point mutation in the fibroblast growth factor receptor 3 (FGFR3) gene, which is located on chromosome 4p16.3 [2, 4, 5]. While the inheritance pattern is autosomal dominant, more than 90% of cases are attributed to de novo mutations [6].

It is often associated with spinal canal stenosis in children, such as craniocervical junction stenosis due to a shortened skull base, thoracolumbar kyphosis due to wedge-shaped vertebrae, and lumbar spinal canal stenosis (LSCS) [7]. In pediatric patients, surgical intervention for spinal stenosis is considered analogous to that in adults. However, the optimal surgical procedure should be selected because concomitant thoracolumbar kyphosis is frequently observed.

This case report not only describes the case of an 11-year-old boy with ACH who required surgical intervention for LSCS but also presents a review of the literature.

## Case presentation

An 11-year-old Asian boy who was diagnosed with ACH at birth presented with bilateral lower limb numbness and intermittent claudication. Lower limb numbness, predominantly on the right side, developed a few years prior; however, bilateral numbness and intermittent claudication that limited his walking distance to 100 m were noted for the past year. He started growth hormone replacement therapy at 3 years of age, with no positive effect on his short stature. No mental or intellectual developmental abnormalities were observed.

## Physical examination

The patient’s weight was 29.2 kg, height was 119.5 cm (−4.26 standard deviation [SD]), and body mass index (BMI) was 23.3 kg/m^2^. The patient was short in stature and had characteristic facial features of ACH, such as an enlarged head circumference and a prominent forehead.

Although muscle strength was not reduced, numbness was observed from the bilateral gluteal region to the lateral side of the lower extremities while in the resting position. No neurological symptoms or abnormalities were observed in the upper extremities. Deep tendon reflexes, the Babinski reflex, and bladder and rectal function were all normal.

## Imaging studies

**Fig. 1:**
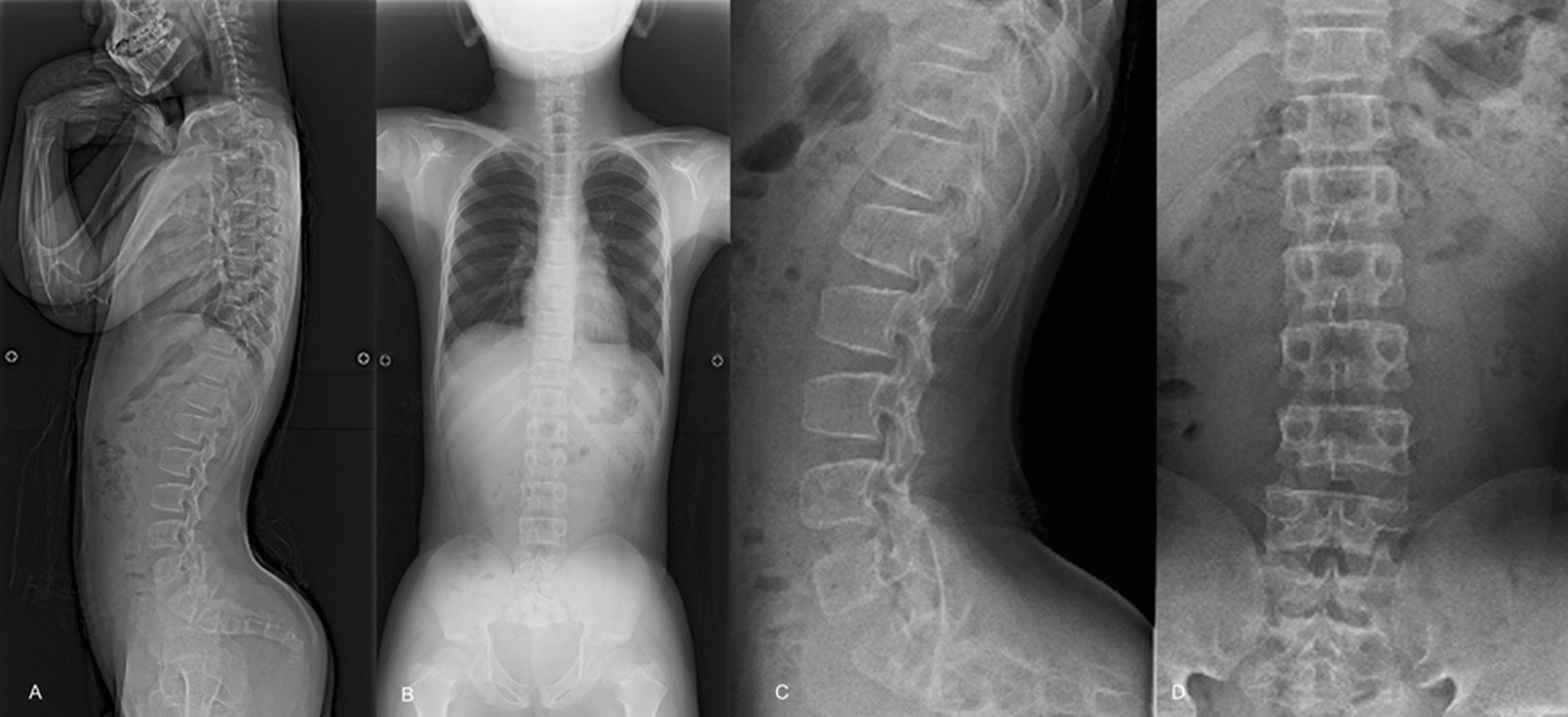
Preoperative whole-spine and lumbar X-ray images (**A**: lateral view of the whole spine, **B**: frontal view of the whole spine, **C**: lateral view of the lumbar spine, **D**: frontal view of the lumbar spine). These images revealed characteristic features of achondroplasia, such as hyperlordosis (L1–S1), a large pelvic tilt angle, and shortened pedicles. However, there was no significant evidence of thoracolumbar kyphosis

**Fig. 2:**
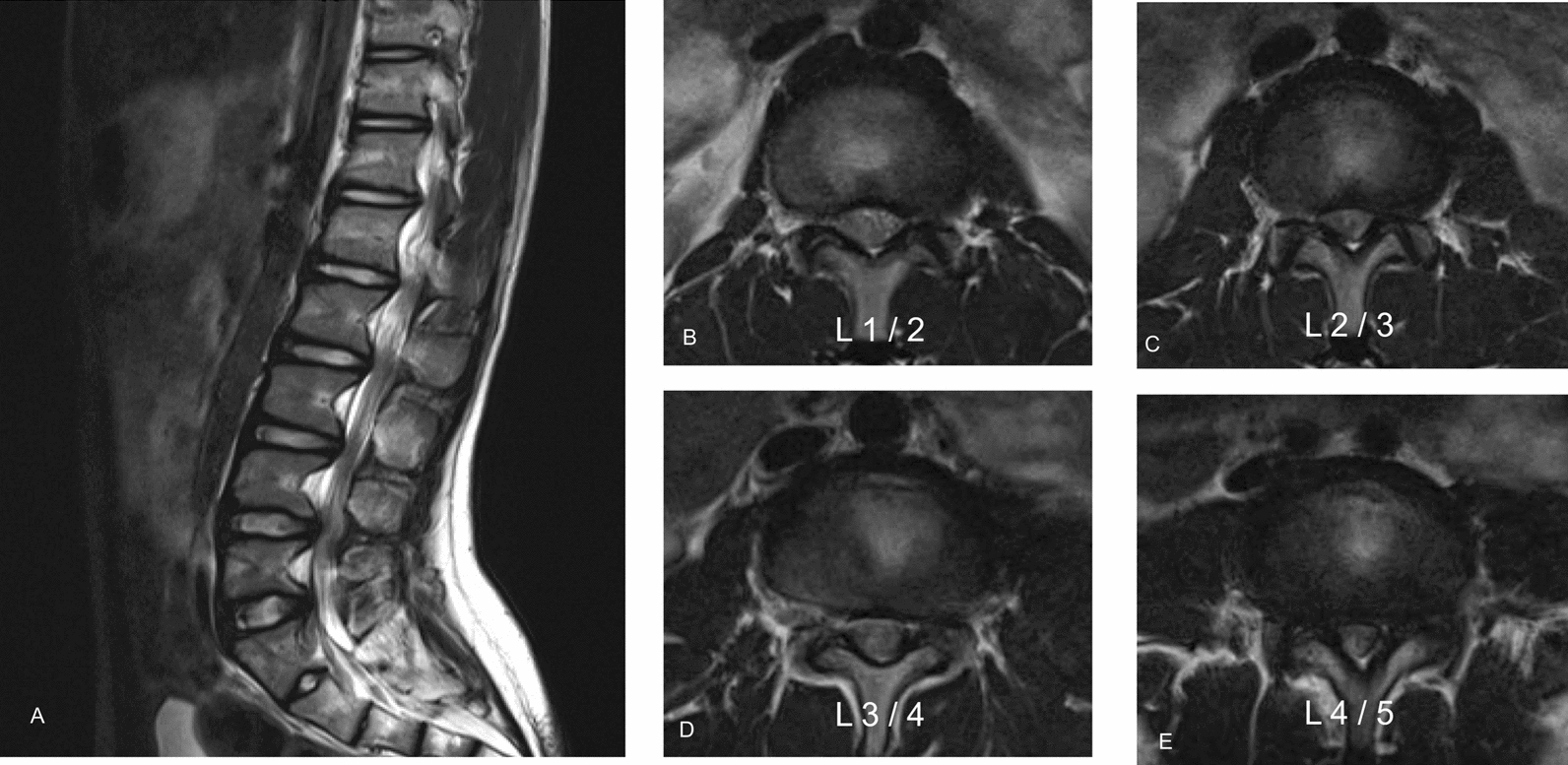
Preoperative magnetic resonance imaging of the thoracolumbar region revealed multilevel stenosis from T12 to S1, with a redundant nerve root sign (**A**: sagittal plane, **B**: transverse plane of L1/2, **C**: transverse plane of L2/3, **D**: transverse plane of L3/4, **E**: transverse plane of L4/5). The most stenosis was observed at the L4/5 level

X-ray images revealed characteristic findings of ACH, such as hyperlordosis of the lumbar spine (L1–S1) and shortened pedicles. Examination of his sagittal alignment revealed the following features: a pelvic incidence (PI) of 45°, a pelvic tilt (PT) of 42°, thoracic kyphosis (TK) of 21°, thoracolumbar kyphosis (TLK) of 5°, and lumbar lordosis (LL) of 55° (Fig. 1). All the parameters corresponding to sagittal alignment were measured on the basis of the methodology described by Schwab *et al*. [8]. Computed tomography (CT) of the spine revealed caudal narrowing of the transverse diameter of the vertebrae of the lumbar spine despite no change in the anteroposterior diameter. Magnetic resonance imaging (MRI) of the entire spine revealed multilevel stenosis from T12 to S1, with a redundant nerve root sign. The most stenosis was observed at the L4/5 level (Fig. 2). No significant stenosis was observed at the craniovertebral junction. The patient presented symptoms consistent with those of cauda equina syndrome, and a diagnosis of spinal canal stenosis associated with pediatric ACH was made.

## Surgical intervention

We planned laminotomy combined with spinous process reconstruction using a mini plate. Given the absence of thoracolumbar kyphosis, we did not consider the need for additional spinal fusion. With the use of mini plates, we chose a reconstructive method specifically designed for posterior lumbar spine structures such as spinous processes, supraspinous ligaments, and interspinous ligament complexes. This method allows for maximum preservation of the midline posterior tension band structure.

**Fig. 3:**
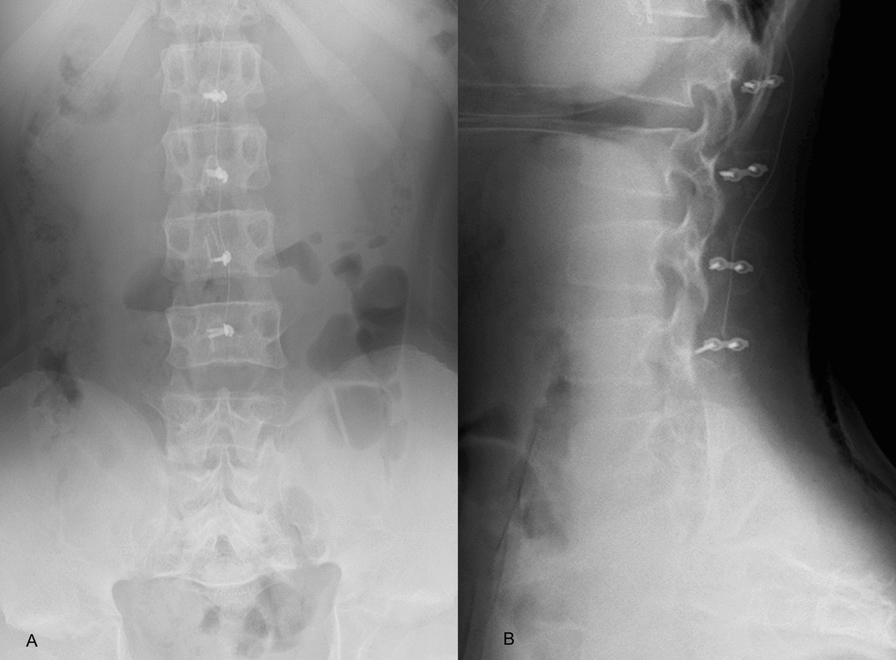
Postoperative lumber X-ray images (**A**: lateral view, **B**: frontal view). Spinous process reduction laminotomy (T12–S1) was performed. The detached spinous processes, except the L5 spinous process, were reattached via mini plate–screw fixation. The L5 spinous process was secured with surgical sutures because the plate was difficult to place because of hyperlordosis

Under O-arm navigation (Medtronic Sofamor Danek, Memphis, USA) guidance and intraoperative neuromonitoring, multilevel laminotomy combined with spinous process reconstruction (T12–S1) was performed. The patient was placed in the supine position under general anesthesia, and a posterior midline longitudinal skin incision was made over the spinous processes from T12 to the sacrum. To preserve the continuity of the supraspinous/interspinous ligament and the spinous processes, a longitudinal incision was made in the fascia of the posterior spinal musculature 5 mm lateral to the midline on both sides. The multifidus muscle was then subperiosteally dissected from the lateral aspect of the spinous processes and the lamina. The spinous processes of the lumbar vertebrae (L1–5) were temporarily detached at the base of each lamina using a Reston bone shear, after which the supraspinous and interspinous ligaments were sharply cut at the L5/S interspinous level. The spinous processes and ligament complex were temporarily flipped cranially to obtain a wide surgical field. Multilevel fenestration (L1–S1) was performed to decompress the dural sac and nerve roots. During posterior decompression, navigation guidance was utilized because the patient’s posterior bony structures were small and thin. No degenerative ligamentum flavum thickening was observed, and minimal epidural fat was present. The dura was thin and translucent. After posterior decompression was complete, the detached spinous processes, except the L5 spinous process, and the ligament complex were reattached using a mini plate screw. The L5 spinous process was secured with surgical sutures because the plate was difficult to place because of lumbosacral hyperlordosis (Fig. 3).

## Postoperative course

**Fig. 4:**
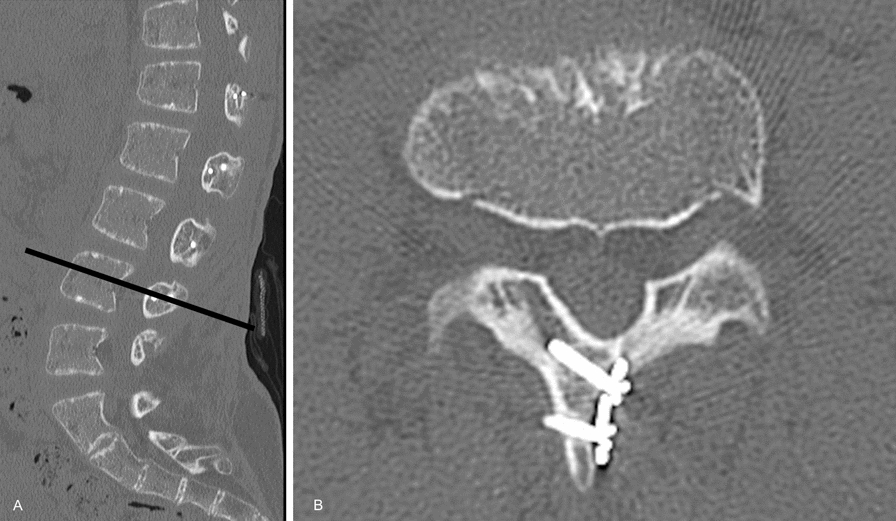
At the 2-year postoperative follow-up, computed tomography images confirmed proper bony fusion at the plate fixation sites (**A**: sagittal plane, **B**: transverse plane)

**Fig. 5:**
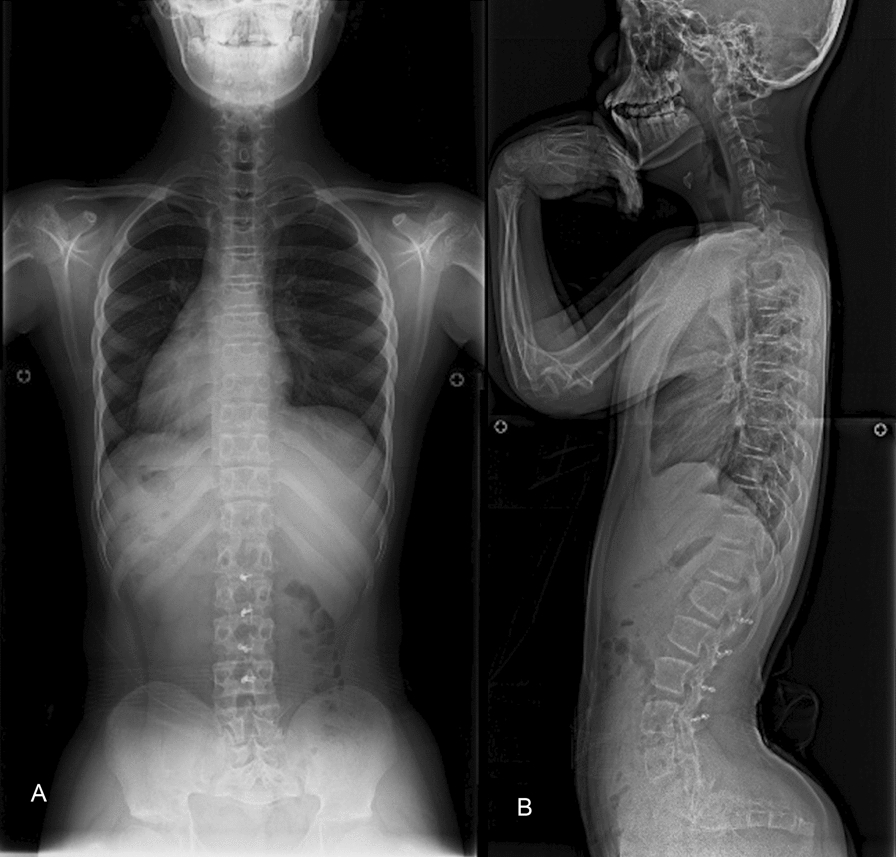
Postoperative whole-spine X-ray images (**A**: lateral view, **B**: frontal view). No remarkable kyphotic deformity or alignment changes were observed

The patient was allowed to ambulate while wearing a soft thoracolumbar orthosis on the first day after surgery. The postoperative course was uneventful, and the patient was discharged home on the eighth postoperative day. Bilateral leg numbness and intermittent claudication resolved immediately after surgery. Postoperative care included activity restrictions and the use of a thoracolumbar orthosis for 1 month. At the 2-year postoperative follow-up, he did not present with symptoms of recurrence, and CT revealed proper bony fusion at the site of plate fixation (Fig. 4). The final follow-up X-ray images revealed LL of 61°, TK of 26°, and TLK of 19°. A mild kyphotic deformity in the thoracolumbar region was observed; however, the sagittal alignment of the spine remained within the normal range (Fig. 5).

## Discussion

Patients with ACH are predisposed to spinal canal stenosis because of their anatomical characteristics. The anteroposterior length of the pedicles is short, as is the interpedicular distance, resulting in a reduced cross-sectional area of the spinal canal [9]. Magnetics resonance imaging (MRI) studies on lumbar spinal stenosis in patients with achondroplasia have confirmed that, unlike in patients with normal spines, where the interpedicular distance widens from L1 to L5, in patients with achondroplasia, this distance progressively narrows from L1 to L5 [9]. This anatomical characteristic, combined with caudal narrowing, is a significant contributing factor to the development of lumbar spinal stenosis in patients with ACH. Scholars have reported that 6.5% of patients with ACH are diagnosed with scoliosis by the age of 20 years and that 17% are diagnosed by the age of 40 years, approximately 40% of whom have already undergone surgery [10]. A study by Daniel *et al*. revealed that while symptomatic stenosis affects patients with achondroplasia of various ages, more than half of symptomatic patients are under 12 years old, suggesting that stenosis is more common in children than previously thought [11]. In this case, the patient presented with symptoms of cauda equina syndrome at age 10 years, highlighting the importance of considering surgical intervention even for pediatric patients.

Thoracolumbar kyphosis, a hallmark feature of ACH, manifests in 90% of infants. While kyphotic deformities generally resolve spontaneously by the age of 3 years, 11% of infants develop recurrence by the age of 10 years, and more than 30% of adults aged 30 years and older have persistent thoracolumbar kyphosis [12]. This deformity frequently progresses to compensatory thoracic hyperlordosis and lumbar hyperlordosis [12]. Severe kyphosis can lead to debilitating low back pain, thigh muscle fatigue and pain, and gastroesophageal reflux-related feeding difficulties.

Thoracolumbar kyphosis is an important concern when surgical treatment for LSCS in pediatric patients with ACH is considered. Surgery performed via a posterior approach and including a decompression procedure may cause the loss of posterior structures, thereby increasing the risk of progressive kyphosis. Ain *et al*. reported progressive thoracolumbar kyphosis after decompression surgery in pediatric patients with ACH [13]. Reports on concomitant spinal fusion have revealed that fusion is necessary if a patient presents with kyphosis of 50 degrees or more and is recommended only if the patient presents with slight kyphosis [14, 15]. These findings suggest that the risk of spinal kyphosis should be appropriately managed if only decompression surgery is planned. In contrast, Vleggeert-Lankamp *et al*. reported no such complications, even in patients with kyphotic deformities greater than 40°, in a cohort of 20 patients. Podkovik *et al*. reported that preserving posterior elements may prevent the development of a postoperative kyphotic deformity [16]. There is no consensus on the need for fusion. However, the addition of long-segment spinal fusion can impose substantial limitations on patients’ ability to perform activities of daily living (ADL). These patients often have short limbs; thus, long-segment spinal fusion should be avoided whenever possible.

In this case, decompression without fusion surgery was performed to preserve or improve the patients’ ability to perform ADLs, as no significant kyphosis was observed. To preserve the midline posterior tension band structure, a spinous process reconstructive fenestration technique was employed, providing excellent visibility of the surgical field and enabling simple posterior element reconstruction. The preserved spinous process and interspinous ligament complex were reattached to the lamina using mini plates (multihand loc system, Bear Medic Corporation, Ibaraki Prefecture, Japan), successfully avoiding extensive spinal fixation. A total of 2 years after surgery, no significant kyphotic deformities or complications were observed, and favorable outcomes were reported.

Surgery in pediatric patients with ACH can be challenging because of their small and thin bony structures, thin and fragile dura, minimal epidural fat, and abnormal spinal canal morphology. In this case, navigation-assisted drilling ensured appropriate decompression without injuring the dura. In addition, intraoperative neuromonitoring enhances surgical safety.

## Conclusion

It is still unclear whether decompression should be combined with spinal fusion in pediatric patients with ACH who present with neurogenic claudication. The benefits of spinal fusion should be weighed against the risks of postoperative development of thoracolumbar kyphosis and adjacent intervertebral disorders in addition to the patient’s likelihood of being limited in performing ADLs.

This case demonstrates that meticulously planned decompression surgery with a focus on preserving the posterior elements, such as multilevel laminotomy with spinous process reconstruction using mini plates, can yield good outcomes for pediatric patients with ACH complicated by LSCS even without long-segment spinal fusion. Nevertheless, long-term follow-up is imperative for monitoring the potential progression of thoracolumbar kyphosis.

## Data Availability

The datasets are available from the corresponding author on reasonable request.

## Abbreviations

ACH: Achondroplasia
LSCS: Lumbar spinal canal stenosis
FGFR3: Fibroblast growth factor receptor 3
PI: Pelvic incidence
PT: Pelvic tilt
TK: Thoracic kyphosis
TLK: Thoracolumbar kyphosis
LL: Lumbar lordosis
CT: Computed tomography
MRI: Magnetic resonance imaging
ADL: Activities of daily living
